# Sero-prevalence of *Helicobacter pylori* Infection in Neyshabur, Iran, During 2010-2015

**Published:** 2017-04-01

**Authors:** Mohammad Salehi, Abdolmajid Ghasemian, Seyyed Khalil Shokouhi Mostafavi, Somayyeh Najafi, Hassan Rajabi Vardanjani

**Affiliations:** 1 *Medical Diagnostic Laboratory of Neyshabour, Center of Medical, Pathological and Genetic Diagnostic Services, Iranian Academic Center for Education, Culture and Research (ACECR), Mashhad Branch, Mashhad, Iran*; 2 *Research Center for HIV/AIDS, HTLV and Viral Hepatitis, Iranian Academic Center for Education, Culture and Research (ACECR), Khorasan Razavi Branch, Mashhad, Iran*; 3 *Microbiology Department, Faculty of Medicine, AJA University of Medical Sciences, Tehran, Iran*; 4 *Dept. of Bacteriology, Faculty of Medical Sciences, Tarbiat Modares University, Tehran, Iran*; 5 *Dept. of Microbiology, Tehran Medical Sciences Branch, Islamic Azad University, Tehran, Iran*; 6 *Dept. of Microbiology, Faculty of Biological Sciences, Islamic Azad University, Tonekabon Branch, Tonekabon, Iran*; 7 *Researcher of Shahrekord University of Medical Sciences, Shahrekord, Iran*

**Keywords:** *Helicobacterpylori*, Seroprevalence, Neyshabur, Enzyme-linked Immuno Sorbent Assay

## Abstract

**Backgrounds & Objective:**

The *Helicobacter pylori* prevalence has continuously decreased during recent years in Iran. The current study aimed at determining *H*. *pylori* prevalence in Neyshabur city, Northeast Iran, during 2010-2015.

**Methods:**

The current epidemiologic survey was conducted in Neyshabur from 2010 to 2015 to determine the prevalence of *H. pylori *infection. A total of 11596 participants (3681 male with the mean age of 31.7±6.2 years and 7915 female with mean age of 68.3±4.7 years) were included. The enzyme-linked immunosorbent assay kits for the detection of *H. pylori *and Stat Fax 3200® Microplate Reader (USA) with a sensitivity of 95% and specificity of 98% were used. Titers above 12 units were considered positive for IgG, IgA, and IgM (negative <8, equivocal 8 to 12, and positive >12 U). The Chi-square *t* test and F test were used to analyze data.

**Results and Conclusion:**

The overall IgA, IgG, and IgM seropositive samples among the study participants were 852 (7.2%), 9000 (72.8%), and 1256 (5.2%), respectively. The IgA seropositivity was significantly high among the age group above 51 years, compared with the other age groups. Moreover, the IgG and IgM seropositivity were significantly high among the age groups 41 to 50 and 31 to 40 years respectively, compared with the other age groups. There was no significant difference between male and female cases regarding IgA and IgG seropositive samples, but IgM level was significantly higher among females, compared with that of the male cases. Furthermore, there was no significant alteration in IgA, IgG, and IgM seropositivity during 2010-2014 in Neyshabur. The prevalence of *H. pylori* in Neyshabur was high in the healthy population. Furthermore, the *H. pylori* prevalence did not change from 2010 to 2014 in the studied city. Effective approaches to improve health, educational, and socioeconomic status should be implemented to minimize and control *H. pylori* infection.

## Introduction

*Helicobacter* *pylori *(*H. pylori*) prevalence varies worldwide; however, it remains higher than 50% in many areas of the world including South America, East Europe and Asia. *Helicobacter pylori* is associated with gastro-intestinal disorders, with important consequences of chronic gastritis, peptic ulcer that leads to gastric cancer, indigestion, and non-ulcer dyspepsia (1). In a review study on the prevalence and risk factors of *H. pylori* infection in the Middle East, it was demonstrated that *H. pylori* infection was high in the region, and in addition, the rate was higher among the patients with dyspepsia, those with histologically confirmed gastritis, and among the older age groups (2). It was determined that the prevalence of *H. pylori* was at the highest level in Turkey and Egypt (>80%), followed by Iran and Oman (70% and 80%, respectively) before 2013. Furthermore, the prevalence of duodenal ulcers, peptic ulcers, and gastritis among *cagA*+ individuals was 53% (95% confidence interval (CI): 20% to 86%), 65% (95%CI, 34% to 97%), and 71% (95%CI, 59% to 84%), respectively (3). OThe *H. pylori* infection transmission mainly occurs via interpersonal route; however, several other ways are suggested and not clearly explained. On the other hand, the prevalence among younger generations was low (4). A review and meta-analytic study showed that the mean prevalence of *H. pylori* was 50.7% during 1994- 2011 with the lowest and highest prevalence in Tehran (19.2%) and Mazandaran (74.27%) provinces, respectively (5). A survey in Tehran showed that 87.6% of patients with dyspepsia were infected with *H. pylori* (6). Another study on 14 860 consecutive patients with gastritis in Tehran from 2008 to 2014 determined that 83.5% of the cases were infected with *H. pylori*. In addition, a decline was observed in its prevalence during recent years (7). Several other studies from Asia and Middle East showed that *H*. *pylori* infection decreased in recent years (8, 9). The current study was performed to determine the prevalence of *H. pylori* infection in Neyshabur, Northeast Iran, during 2011-2015. 

## Materials and Methods

**Sera samples**

The current epidemiologic, descriptive, cross sectional study was performed in Neyshabur, Northeast Iran, from 2010 to 2015 to determine the prevalence of *H. pylori *infection. A total of 11 596 participants referred to the referral laboratory of Mashhad were included and sera samples (a 5-mL blood sample) were prepared and stored at -20ºC until use.

**ELISA test**

 The enzyme-linked immunosorbent assay (ELISA) kits for the detection of *H. pylori *(Pishtaz teb) and a Stat Fax 3200® Microplate Reader (USA) with the sensitivity of 95% and specificity of 98% were used. Any titer above 12 units was considered positive for IgG, IgA, and IgM (negative <8, equivocal 8 to 12, and positive >12 U).

**Ethical Approval**

The current study protocol was approved by the Research and Technology, Deputy of Iranian Academic Center for Education, Culture, and Research (ACECR), Mashhad Branch. 

**Data Analysis**

Data were analyzed by SPSS version 20 (IBM SPSS Statistics for Windows, Version 20, Armonk, NY, IBM Corp.); the Chi-square and F tests were employed. P-values <0.05 and F <0.001 were considered statistically significant. 

## Results

**Fig 1:**
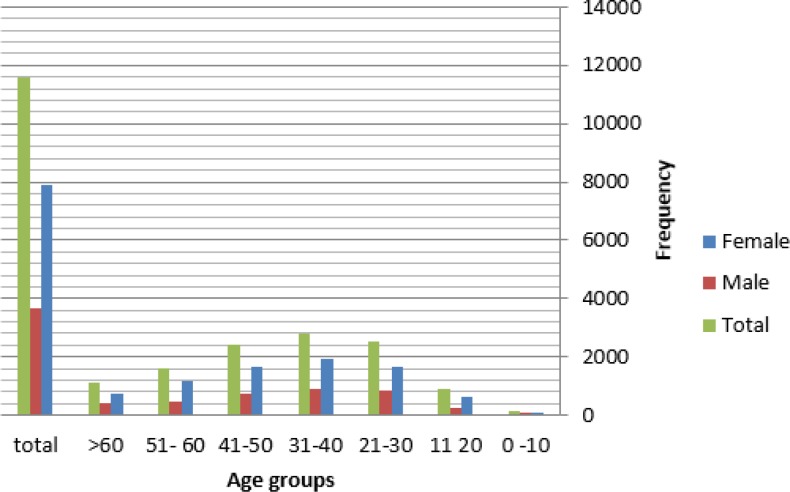
Age-based Distribution of Healthy Individuals in the Study

**Fig 2:**
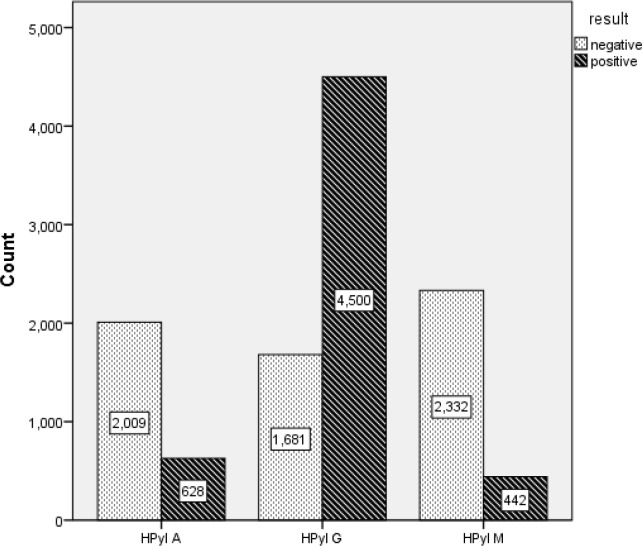
Number of IgA, IgG, and IgM Seropositive Cases of the Study

 Out of 11 596 participants, 3681 were male and 7916 female. The mean age of the cases was 45.39 ±15.4 years. Overall, 4500 (38.8%) cases indicated evidence of IgG seropositivity against *H. pylori *(ELISA IgG-Ab+), followed by 854 (7.3%) cases for IgA and 600 (5.2%) for IgM. The levels of IgG antibody from 2010 to 2015 were 73.6%, 67.9%, 72.4%, 75.6%, and 75.4%, respectively. These levels showed no significant or sensible change during the years of study, using F test (Pr>F, F>0.001). The IgA seropositivity was significantly high among the age group above 51 years, compared with the other age groups (F<0.001). Moreover, the IgG and IgM seropositivity were significantly high among the age groups 41 to 50 and 31 to 40 years, respectively (F<0.001). There was no significant difference between male and female cases regarding IgA and IgG seropositivity, but females had a significantly higher rate for IgM seropositivity, compared with that of the male cases (4.2 vs. 2.5, P=0.044). Furthermore, there was no significant alteration in IgA, IgG, and IgM seropositivity during 2011-2014 (tables 1 and 2, Pr>F, F>0.001). Figures 1 and 2 show the level of antibodies and serum level (%) of antibodies, respectively. 

**Table 1:** The Relationship Between Antibody Titer, Age, and Gender - 2010-2015

P-value	95% Cl			Odds Ratio	Seropositivity, N (%)			No. (%)	Demographic Features	
					IgM	IgG	IgA			
Baseline					2 (1.3)	30 (19.3)	1 (0.6)	155(1.3)	0-10	**A** **ge group (year)**
**0.002**		1.321-3.398	2.119		43 (4.71)	265 (29.1)	10(1.1)	912(7.9)	11-20	
**<0.0001**		2.864-7.104	4.511		108 (4.27)	959 (37.9)	85 (3.36)	2527(21.8)	21-30	
**<0.0001**		4.190-10.424	6.609		119 (4.22)	1155 (41)	140 (4.97)	2817(24.3)	31-40	
**<0.0001**		4.589-11.495	7.263		93 (3.82)	1022 (42)	149 (6.1)	2435(21)	41-50	
**<0.0001**		4.582-11.728	7.33		53 (3.3)	656 (40.4)	131 (8.1)	1623(14)	51-60	
**<0.0001**		3.201-8.296	5.153		24 (2.1)	413 (36.6)	112 (9.9)	1127(9.7)	61<	
**0.653**		0.908-1.062	0.982		95 (2.5)	1440 (39.1)	222 (6)	3681(31.7)	Male	**Gender**
					347 (4.4)	3060 (38.7)	406 (5.13)	7915 (68.3)	Female	
					**442 (7.2)**	**4500(38.8)**	**628(5.3)**	**11596 (100)**		**Total**

**Table 2:** The Serum Levels of IgG and IgA against *Helicobacter* *pylori* During 2011-2014

Year	No.	IgG Seropositivity, N (%)	IgG Seronegativity, N (%)
2014	762	575 (75.4)	187 (24.5)
2013	1297	980 (75.6)	317 (24.4)
2012	1195	866 (72.4)	329 (27.5)
2011	1348	916 (67.9)	432 (32)
2010	1583	1166 (73.6)	417 (26.3)
Total	**6185**	**4500 (72.8)**	**1682 (27.2)**

It was the first study on *H. pylori* prevalence in Neyshabur, Northeastern Iran. Of the 11 596 participants in the current study, 1681 (78.2%) had the evidence of IgG seropositivity, followed by 854 (7.3%) cases for IgA and 600 (5.2%) for IgM seropositivity against *H. pylori *using ELISA kits. The levels of IgG antibody from 2010 to 2015 were 73.6%, 67.9%, 72.5%, 75.6%, and 75.4%, respectively. These levels showed no significant or sensible change during the years of study. However, most of the previous studies showed that *H. pylori* prevalence gradually decreased in recent years in some areas of Iran and several other countries and that is increased with age (4, 5, 10-12). A study by Ashtari on 14 860 patients revealed that 83.5% of the cases were infected with *H*. *pylori*. Moreover, the prevalence of bacteria in severe gastritis was significantly (P˂0.05) higher than those of mild or moderate statuses. It was concluded that its rate was high among Iranian population, but declined during recent years (7). Moreover, as depicted in Table1, the overall IgA, IgG, and IgM seropositive samples among different age groups of the current study were 442 (3.81%), 4500 (38.8%), and 628 (2.31%), respectively. The IgA seropositivity was significantly high among the age group above 51 years. Moreover, the IgG and IgM seropositivity were significantly high among the age groups 41 to 50 and 31 to 40 years, respectively. There was no significant difference between male and female cases regarding IgA and IgG seropositivity, but females had a significantly higher rate for IgM seropositivity, compared with that of male cases. Furthermore, there was no significant alteration in IgA, IgG, and IgM seropositivity from 2011 to 2014. In a study by Nouraie on 2561 healthy individuals, the prevalence of *H*. *pylori* was 69% and was correlated with age increase in Tehran province, Iran (13). In another study by Alborzi, in Southern Iran, the prevalence of *H*. *pylori* were 82%, 98%, 88%, 89%, and 58% among 9-month-, and 2-, 6-, 10-, and 15-year-old cases, respectively, in which there was a significant decrease among the 15-year-old group (14). Moreover, gastric cancer, the most common type of cancer in the North and Northwest Iran, was also highly related to *H. pylori *infection, indicating a high prevalence of *H. pylori* in these areas (15). In a systematic review and meta-analytic study by Eshraghian, the prevalence of *H. pylori* ranged from 22% to 87% among healthy populations in Iran and the other Eastern Mediterranean region countries (9). In another study by Khedmat, among the Middle Eastern countries, the prevalence of *H. pylori* was high and the rate seemed to be higher among patients with dyspepsia, patients with histologically confirmed gastritis, and also in patients of the elderly groups. Furthermore, Turkey and Egypt showed the highest rate of infection (>80%), in Iran and Oman it was 70% and 80%, respectively (2). On the other hand, it was revealed that the *H. pylori* prevalence was high among less economically advanced and populated countries. Moreover, the intrafamilial transmission may occur. *Helicobacter pylori* transmission through water showed controversial results (8). In the present study, the relationship of *H*. *pylori* IgA and IgG prevalence with age increase of individuals was significant (Table 1), but not for different genders. The rate of IgA, IgG, and IgM seropositivity among the age group 0 to 10 years was 0.6%, 19.3%, and 1.3%, respectively, as the lowest level. The highest serum level of antibodies was observed among the age groups >6, 41 to 50, and 21 to 30 years old, respectively. Furthermore, the slight alteration in the level of IgG, IgA, and IgM antibodies during 2011-2014 was insignificant. 

## Conclusion

 The prevalence of *H. pylori* in Neyshabur was high among the healthy population. Furthermore, the *H*. *pylori* prevalence did not change from 2010 to 2014 in the studied city. These results highlighted the high risk among all of ages groups and possibility of transmission of the disease in this area. IgA seropositivity was significantly high among the age group above 51 years, while the IgG and IgM seropositivity were significantly high among the age groups 41 to 50 and 31to 40 years, respectively. IgM level was significantly higher among females, but not in case of IgA and IgG. Effective approaches to improve sanitary purposes, and educational and socioeconomic status should be implemented to minimize and control *H. pylori* infection. 
